# Efficacy and safety of tislelizumab in patients with advanced esophageal squamous cell carcinoma: a systematic review and meta-analysis

**DOI:** 10.1186/s43046-025-00315-w

**Published:** 2025-08-25

**Authors:** Eric Ricardo Yonatan, Surya Sinaga Immanuel, Erlangga Saputra Arifin, Louis Fabio Jonathan Jusni, Riki Tenggara, Mario Steffanus, Delia Anastasia Tirtadjaja

**Affiliations:** 1 School of Medicine and Health Sciences, Atma Jaya Catholic University of Indonesia, Jakarta, Indonesia; 2 Department of Internal Medicine, School of Medicine and Health Sciences, Atma Jaya Catholic University of Indonesia, Jakarta, Indonesia

**Keywords:** Tislelizumab, Advanced esophageal squamous cell carcinoma, Survival analysis, Treatment outcome, Adverse events

## Abstract

**Background:**

Tislelizumab, a PD-1-targeting monoclonal antibody, can potentially treat advanced esophageal squamous cell carcinoma (ESCC). Using pooled clinical data, this study evaluates Tislelizumab's efficacy and safety in advanced ESCC.

**Methods:**

This review followed PRISMA guidelines, with a comprehensive search conducted across PubMed, ProQuest, EBSCOhost, and Google Scholar for clinical trials involving ESCC patients treated with Tislelizumab. Primary endpoints included overall survival (OS), progression-free survival (PFS), objective response rate (ORR), disease control rate (DCR), and adverse events (AEs). We evaluated the study quality using the Cochrane Risk of Bias and ROBINS-I tools. Data extraction and pooling were performed using R for single-arm studies and RevMan 5.4 for RCTs. Outcomes were analyzed using pooled medians, hazard ratios for OS and PFS, and relative risks for ORR, DCR, and adverse events.

**Results:**

Four studies with 1,202 patients met inclusion criteria. The risk of bias was low to moderate. Pooled data indicate a median OS of 8.6 months and PFS of 4.75 months in the Tislelizumab group, with an overall ORR of 0.40 (95% CI: 0.20–0.61) and DCR of 0.64 (95% CI: 0.36–0.88). Tislelizumab significantly improved OS (HR 0.68, 95% CI: 0.59–0.78, *p* < 0.0001), PFS (HR 0.71, 95% CI: 0.54–0.93, *p* = 0.01), ORR (RR 1.65, 95% CI: 1.22–2.24, *p* = 0.001), and DCR (RR 1.11, 95% CI: 1.04–1.18, *p* = 0.001) compared to standard chemotherapy. Pooled rates of grade 3 or more AEs and serious AEs were 0.56 (95% CI: 0.17–0.92) and 0.28 (95% CI: 0.10–0.50), respectively. There were no significant differences in grade 3 and serious AEs between Tislelizumab and standard chemotherapy. The most common AEs reported included hematologic toxicities, gastrointestinal issues, metabolic disturbances, and biochemical abnormalities.

**Conclusion:**

Tislelizumab improves survival and response in advanced ESCC patients, particularly when combined with chemotherapy, with an acceptable safety profile. These findings support its continued use in ESCC, though further investigation is warranted due to the limited number of studies.

**Trial registration:**

CRD42024564367.

## Background

Esophageal squamous cell carcinoma (ESCC) is a significant global health concern, ranking as the seventh most commonly diagnosed cancer worldwide. In 2020, approximately 604,100 new cases and 544,100 deaths were attributed to esophageal cancer, with ESCC accounting for about 85% of these cases [1]. The high mortality rate is due primarily to late-stage diagnoses and the limited efficacy of current treatment options.

The management of ESCC poses a substantial clinical challenge, primarily due to delayed diagnosis, poor prognosis, and limited therapeutic options. Early detection is frequently impeded by nonspecific symptoms, such as mild dysphagia or unintentional weight loss, which are often underestimated or misattributed to benign conditions. Consequently, most patients present with advanced-stage disease at diagnosis [2]. The prognosis in such cases remains poor, with the five-year survival rate consistently below 20% [3]. Current standard treatment modalities are associated with critical limitations, including chemoresistance and short-lived therapeutic responses. Although many patients initially respond to chemotherapy, these responses are often temporary, and rapid disease progression is typical [4]. The emergence of chemoresistance further diminishes the effectiveness of subsequent treatment lines, while the toxicity of available regimens often compromises their tolerability and limits their continued use [5, 6].

Current standard treatments for advanced ESCC vary based on programmed death-ligand 1 (PD-L1) sensitivity. For PD-L1-negative patients, platinum–fluoropyrimidine doublet chemotherapy remains the primary approach. In contrast, PD-L1-positive patients are treated with a combination of standard chemotherapy and PD-L1 inhibitors such as pembrolizumab or nivolumab [7, 8]. Despite these interventions, survival outcomes have not significantly improved, underscoring the urgent need for more effective therapies.

Tislelizumab, a monoclonal antibody targeting programmed cell death protein 1 (PD-1), has emerged as a promising therapeutic agent. Approved by the Food and Drug Administration (FDA) for various PD-L1-sensitive cancers, including non-small cell lung carcinoma, hepatocellular carcinoma, gastric cancer, and urothelial carcinoma, tislelizumab is engineered to reduce immunogenicity and minimize adverse reactions compared to other PD-1 inhibitors. Preliminary studies suggest that tislelizumab may offer an improved prognosis for patients with ESCC; however, comprehensive evaluations of its efficacy and safety are still lacking [9].

This systematic review and meta-analysis aim to assess the efficacy and safety of tislelizumab in patients with advanced ESCC by analyzing data from available clinical trials. By evaluating key outcomes such as overall survival, progression-free survival, and adverse events, we seek to provide critical insights into the potential role of tislelizumab in the treatment landscape of advanced ESCC.

## Methods

This systematic review and meta-analysis followed the Preferred Reporting Items for Systematic Reviews and Meta-Analyses (PRISMA) 2020 guidelines [10]. The review protocol was registered with the International Prospective Register of Systematic Reviews (PROSPERO) under CRD42024564367.

### Eligibility criteria

#### Types of studies

We included all clinical trials (randomized controlled trials or RCTs and non-randomized studies) that evaluated the efficacy and safety of tislelizumab in patients with advanced ESCC. Studies were required to report on at least one of the predefined outcomes of interest. Observational studies, case reports, case series, reviews, conference abstracts, book sections, commentaries, editorials, and studies involving non-human subjects were excluded. Only articles published in English with accessible full-text manuscripts were considered.

#### Participants

Studies enrolling adult patients (≥ 18 years) with histologically confirmed advanced or metastatic ESCC were included. Eligible patients were required to have an Eastern Cooperative Oncology Group Performance Status (ECOG PS) of 0–1 and measurable or evaluable disease according to the Response Evaluation Criteria in Solid Tumors (RECIST) version 1.1. Patients previously treated with monoclonal antibodies against PD-1 were excluded, as were those with active autoimmune diseases or receiving systemic corticosteroids or immunosuppressants.

#### Outcome of interest

The primary outcomes were overall survival (OS), progression-free survival (PFS), objective response rate (ORR), disease control rate (DCR), and the incidence of grade 3 or higher adverse events and serious adverse events.

#### Search strategy

A comprehensive literature search was conducted to identify relevant studies published up to September 30, 2024. The following electronic databases were searched: PubMed, EBSCOhost, ProQuest, and Google Scholar. The search terms used included both keywords and Medical Subject Headings (MeSH): “tislelizumab” AND (“esophageal squamous cell carcinoma” OR “ESCC”). The search strategy was tailored to each database, and Boolean operators effectively combined search terms. No restrictions were placed on the publication date.

Grey literature, such as unpublished studies and conference proceedings, was not included. Reference lists of all retrieved articles were manually screened to identify additional relevant studies. Only articles published in English were considered due to resource limitations for translation.

#### Study selection

All retrieved records were imported into EndNote reference management software, and duplicates were removed. Two independent reviewers screened the titles and abstracts of the identified studies for eligibility. Studies that met the inclusion criteria or lacked sufficient information in the abstract were retrieved in full text for detailed assessment. The full texts were independently reviewed against the predefined eligibility criteria. Any disagreements were resolved through discussion, and if consensus was not achieved, a third reviewer was consulted.

#### Data extraction

Two reviewers extracted data independently using a standardized data extraction form developed for this study. Extracted information included:

- Study Characteristics: First author, year of publication, country, study design, sample size.
- Participant Characteristics: Median age, race, and gender distribution.
- Intervention Details: Dosage and administration protocol of tislelizumab, use of combination therapies.
- Comparator Details (for controlled trials): Type of control treatment (standard chemotherapy).
- Outcomes: OS, PFS, ORR, DCR, incidence of adverse events (grade 3 or higher, serious adverse events).

Discrepancies in data extraction were resolved through discussion.

#### Risk of bias assessment

Two reviewers independently assessed the methodological quality of the included studies.

- Randomized Controlled Trials: The Cochrane Collaboration’s Risk of Bias tool version 2 (RoB 2) was used to evaluate five domains: randomization process, deviations from intended interventions, missing outcome data, measurement of outcomes, and selection of reported results. Each domain was rated as “low risk,” “some concerns,” or “high risk” of bias [11].
- Non-Randomized Studies: The Risk Of Bias In Non-randomized Studies of Interventions (ROBINS-I) tool was applied, assessing seven domains: confounding, participant selection, classification of interventions, deviations from intended interventions, missing data, measurement of outcomes, and selection of reported results. Each domain was rated as “low,” “moderate,” “serious,” or “critical” risk of bias [12].

Any disagreements were resolved through discussion, and consensus was reached among all reviewers.

#### Data synthesis and statistical analysis

For single-arm studies reporting median OS and PFS, we used the 'meta median' package in R (version 4.4.1) to pool medians using appropriate statistical methods for survival data. To stabilize variances, ORR, DCR, and adverse event rates were pooled using the 'meta' package with the Inverse method and the Freeman-Tukey double arcsine transformation.

For RCTs comparing tislelizumab (with or without chemotherapy) to control treatments, data were analyzed using Review Manager (RevMan) version 5.4. Hazard ratios (HRs) with 95% confidence intervals (CIs) were calculated for time-to-event outcomes (OS and PFS) using the inverse variance method. For dichotomous outcomes (ORR, DCR, adverse events), relative risks (RRs) with 95% CIs were calculated using the Mantel–Haenszel method.

A random-effects model was employed for all meta-analyses to account for variability among studies due to differences in study populations, interventions, and outcome measurements. Statistical heterogeneity was assessed using the chi-squared test and quantified with the I^2^ statistic. An I^2^ value of 25% or less indicated low heterogeneity, 26% to 50% moderate heterogeneity, and greater than 50% high heterogeneity [10].

## Results

**Fig. 1:**
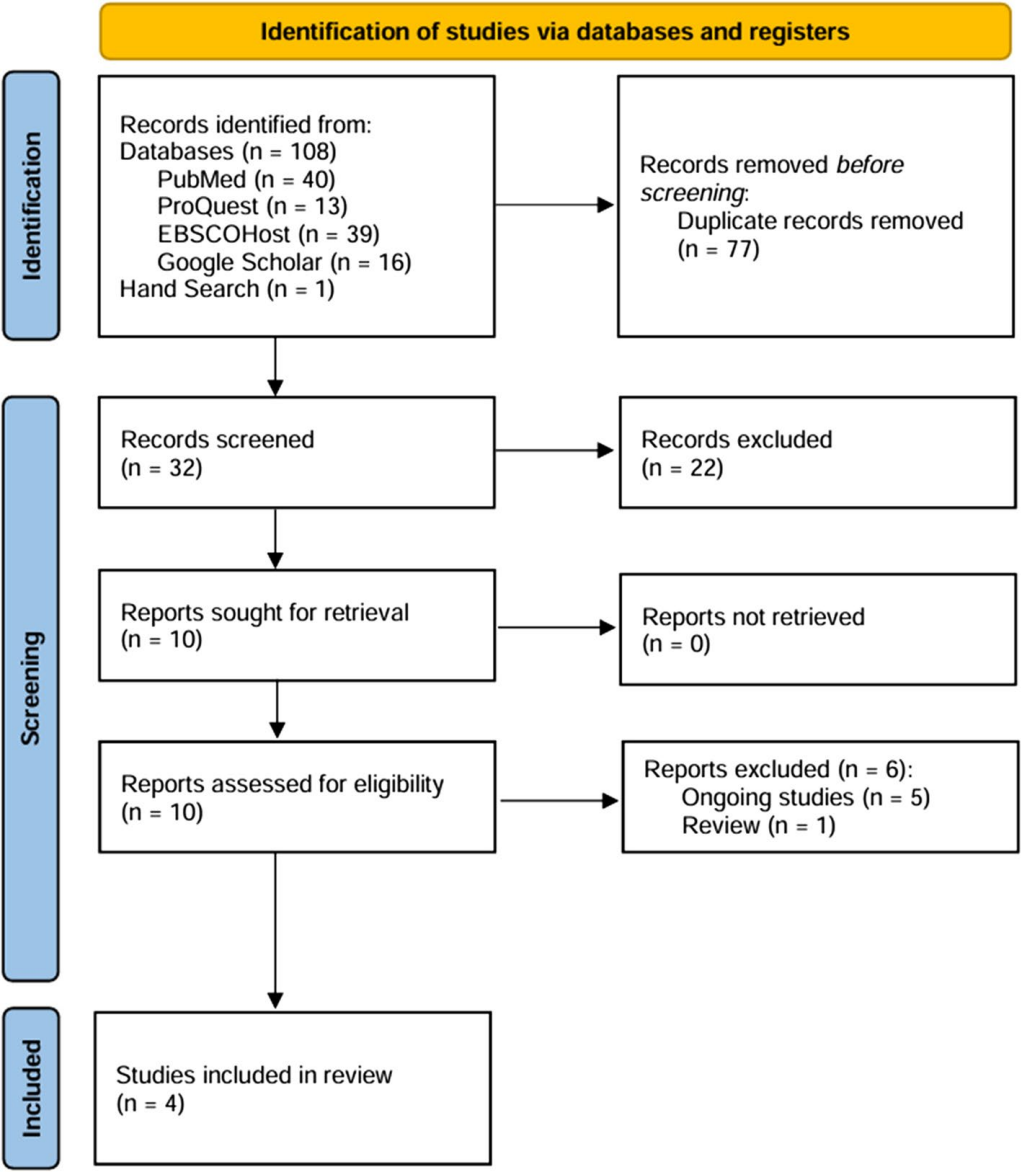
PRISMA 2020 flow diagram of the study selection process

The study selection process is illustrated in the PRISMA 2020 flow diagram (Fig. 1). A total of 108 records were identified through database searching. After removing duplicates, 32 studies remained for the title and abstract screening. Based on the eligibility criteria, 22 studies were excluded at this stage. The full texts of the remaining ten studies were assessed for eligibility, leading to the exclusion of 6 studies due to reasons such as non-relevance to the outcomes of interest or inadequate data. Four studies met the inclusion criteria and were incorporated into the systematic review and meta-analysis.

### Study characteristics

The four included studies encompassed a total of 1,202 patients with advanced ESCC. Key characteristics of these studies are summarized in Table 1. The median age of participants ranged from 61 to 65 years, and the majority were male (85.9%), with females constituting 14.1%. Two studies were multinational, while the others were conducted exclusively in China. Ethnically, 77.8% of patients were Asian, and 21% were White.

**Table 1:** Characteristics of included studies

Study ID	Country	Design	Inclusion Criteria	Group	Participants				Duration	Outcome of Interest
					Total(*n*)	Age (Median, IQR)	Male: Female	Race		
Xu 2023 [13]	Global (Asia, Europe, Oceania, and North America)	Phase III, randomized, double-blind, placebo-controlled trial	Patients aged ≥ 18 years with unresectable, locally advanced, recurrent or metastatic ESCC, ECOG PS of 0–1, and measurable or evaluable disease per RECIST v1.1	Tislelizumab 200 mg IV every 3 weeks with an investigator-chosen chemotherapy doublet: Cisplatin or Oxaliplatin + Fluoropyrimidine (Fluorouracil or Capecitabine) or Paclitaxel.^a^	326	64.0 (59.0–68.0)	282: 44	Asian (75%), White (24%), North America and Oceania (1%)	Treatment continued until disease progression per RECIST 1.1, unacceptable toxicity, death, or withdrawal of consent	OS, PFS, ORR, DCR, AE
				Placebo with an investigator-chosen chemotherapy doublet.^a^	323	65.0 (58.0–70.0)	281: 42	Asian (75%), White (24%), North America and Oceania (1%)		
Xu 2020 [14]	China	Phase II, multicohort clinical trial	Patients aged 18–75 years with pathologically confirmed ESCC, with evaluable lesion considered to be inoperable, locally advanced, or metastatic, have an ECOG PS of 0–1, and a life expectancy ≥ 12 weeks	Tislelizumab 200 mg IV every 3 weeks with cisplatin 80 mg/m2 IV on day 1, and Fluorouracil 800 mg/m2/day IV on days 1–5, in a 21-day cycle	15	61.0 (47.0–68.0)	14: 1	NA	Treatment continued for up to 6 cycles for cisplatin and Fluorouracil and until disease progression, intolerable toxicity, or treatment discontinuation due to any other reason for tislelizumab	PFS, ORR, DCR, AE
Shen 2022 [15]	Global (Belgium, China, France, Germany, Italy, Japan, Republic of Korea, Spain, Taiwan, United Kingdom, United States)	Phase III, randomized, open-label trial	Patients aged ≥ 18 years with histologically confirmed advanced or metastatic ESCC, ECOG PS of 0–1, at least one measurable/evaluable lesion by RECIST v1.1, and adequate hematologic, hepatic, renal, and coagulation function	Tislelizumab 200 mg IV every 3 weeks	256	62.0 (40–86)	217: 39	Asian (78.5%), White (20.7%), Not reported (0.8%)	Treatment continued until disease progression, unacceptable toxicity, or withdrawal for other reasons	OS, PFS, ORR, DCR, AE
				Investigator-chosen single-agent chemotherapies: paclitaxel, docetaxel, or irinotecan.^b^	256	63.0 (35–81)	215: 41	Asian (80.9%), White (17.2%), African-American (0.8%), Other (0.4%), Not reported (0.8%)		
Shen 2020 [16]	China	Phase I/II, open-label, non-comparative study	Patients aged ≥ 18 years with histologically or cytologically confirmed advanced or metastatic solid tumors with measurable disease defined by RECIST v1.1, ECOG PS of 0–1	Tislelizumab 200 mg IV every 3 weeks	26	63.0 (44–77)	23: 3	NA	Treatment continued until patients had no evidence of continued clinical benefit, unacceptable toxicity, or withdrawal of consent	OS, PFS, ORR, DCR, AE

*Abbreviations*: *ESCC* Esophageal squamous cell carcinoma, *ECOG PS* Eastern Cooperative Oncology Group Performance Status, *RECIST* Response evaluation criteria in solid tumours, *IV* Intravenous, *OS* Overall survival, *PFS* Progression-free survival, *ORR* Objective response rate, *DCR* Disease control rate, *AE* Adverse events, *NA* Not available, *MPR* Major pathological response, *pCR* Progression complete response

^a^ Cisplatin 60–80 mg/m^2^ IV on day 1 or Oxaliplatin 130 mg/m^2^ IV on day 1, combined with fluoropyrimidine (fluorouracil 750–800 mg/m^2^ IV on days 1–5 or capecitabine 1000 mg/m^2^ orally twice daily on days 1–14) or paclitaxel 175 mg/m^2^ IV on day 1

^b^ Paclitaxel 135–175 mg/m^2^ IV once every 3 weeks or 80–100 mg/m^2^ once weekly, or Docetaxel 75 mg/m^2^ IV once every 3 weeks, or Irinotecan 125 mg/m^2^ IV on days 1 and 8, every 3 weeks

Regarding study design, two were RCTs, and two were non-randomized studies. The intervention protocols varied slightly but generally involved administering tislelizumab at a dose of 200 mg intravenously every three weeks, either as monotherapy or in combination with investigator-chosen chemotherapy. Control groups in the RCTs received standard chemotherapy following the same schedule. All studies reported the primary outcomes of OS, PFS, ORR, DCR, and safety profiles.

### Quality assessment

**Fig. 2:**
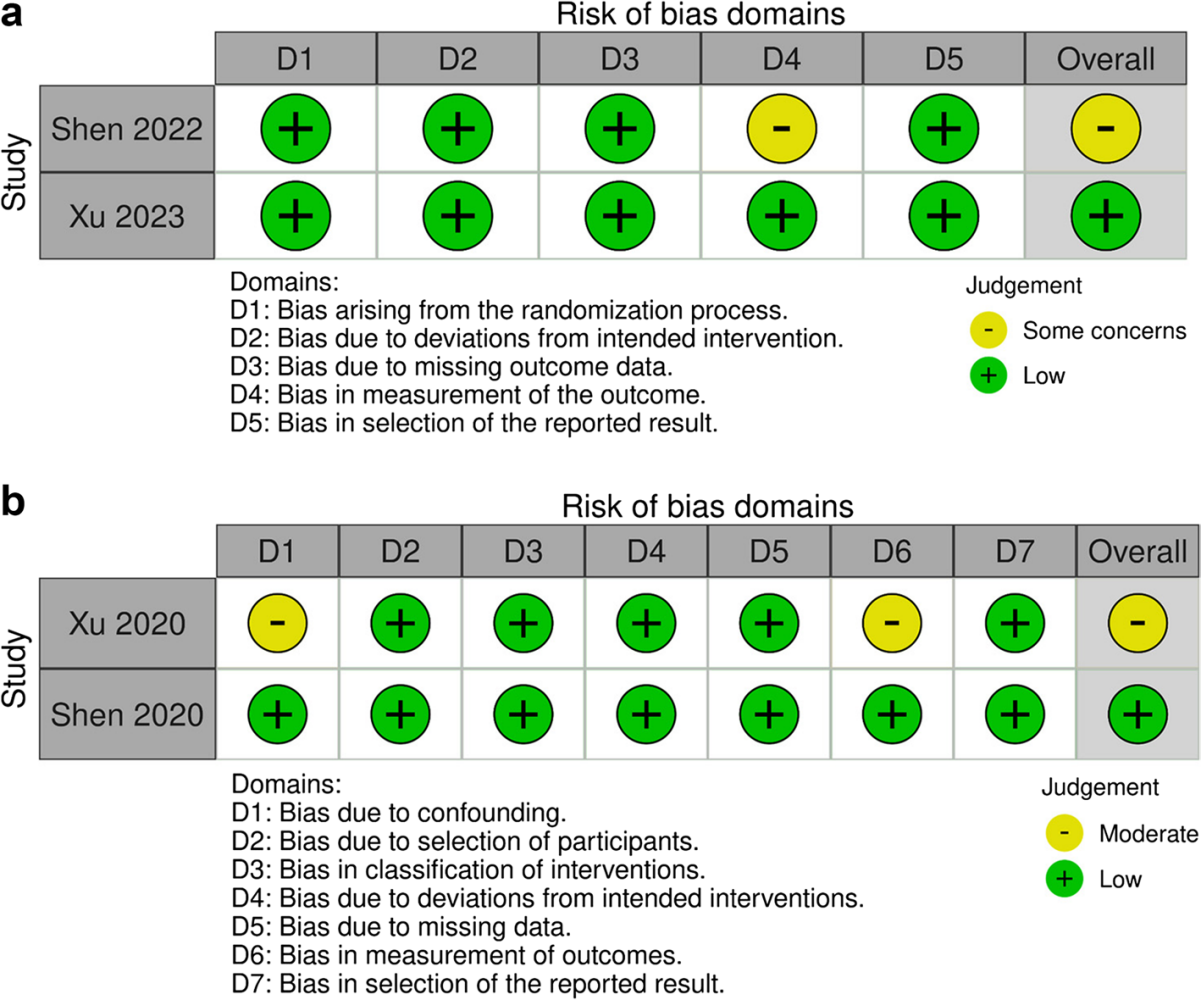
**a** Risk of bias summary for randomized controlled trials using the RoB 2 tool. **b** Risk of bias summary for non-randomized studies using the ROBINS-I tool

Quality assessment was performed using the Cochrane Collaboration tools appropriate for each study design. The two RCTs were evaluated with the Risk of Bias 2 (RoB 2) tool, and both generally had a low risk of bias, though one domain raised some concerns in one study (Fig. 2a). The non-randomized studies were assessed using the Risk Of Bias In Non-randomized Studies of Interventions (ROBINS-I) tool. One study had a moderate risk of bias due to potential confounding factors and selection bias, while the other was rated as low risk overall (Fig. 2b). No studies were excluded based on quality assessment results.

### Quantitative synthesis

**Fig. 3:**
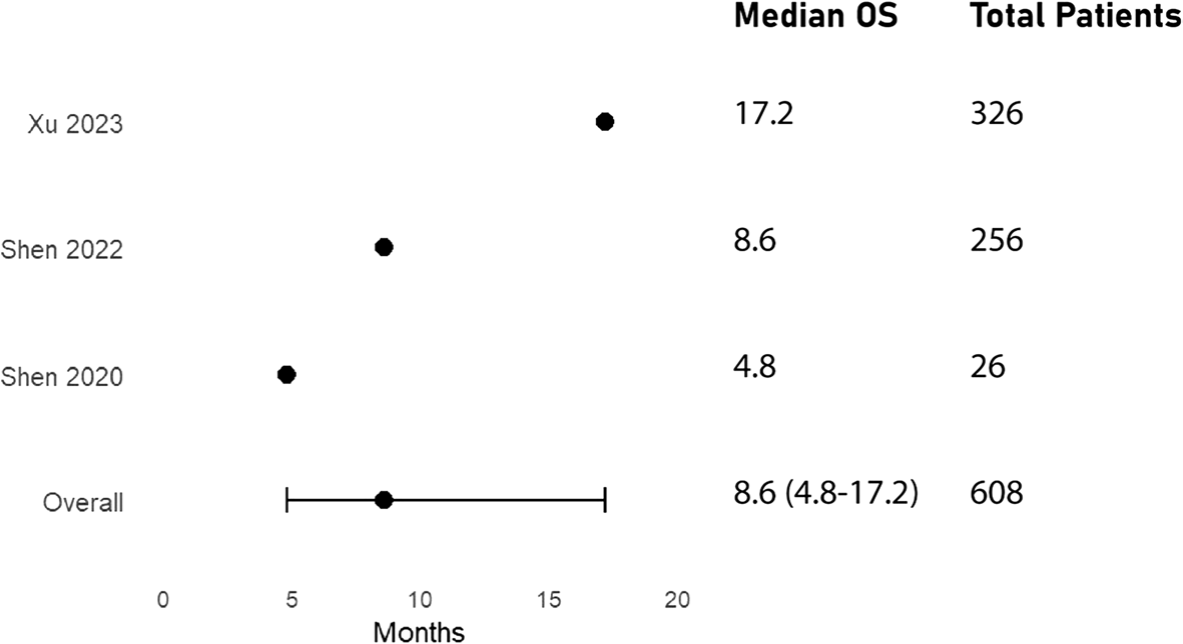
Pooled median overall survival (OS) for patients receiving tislelizumab

**Fig. 4:**
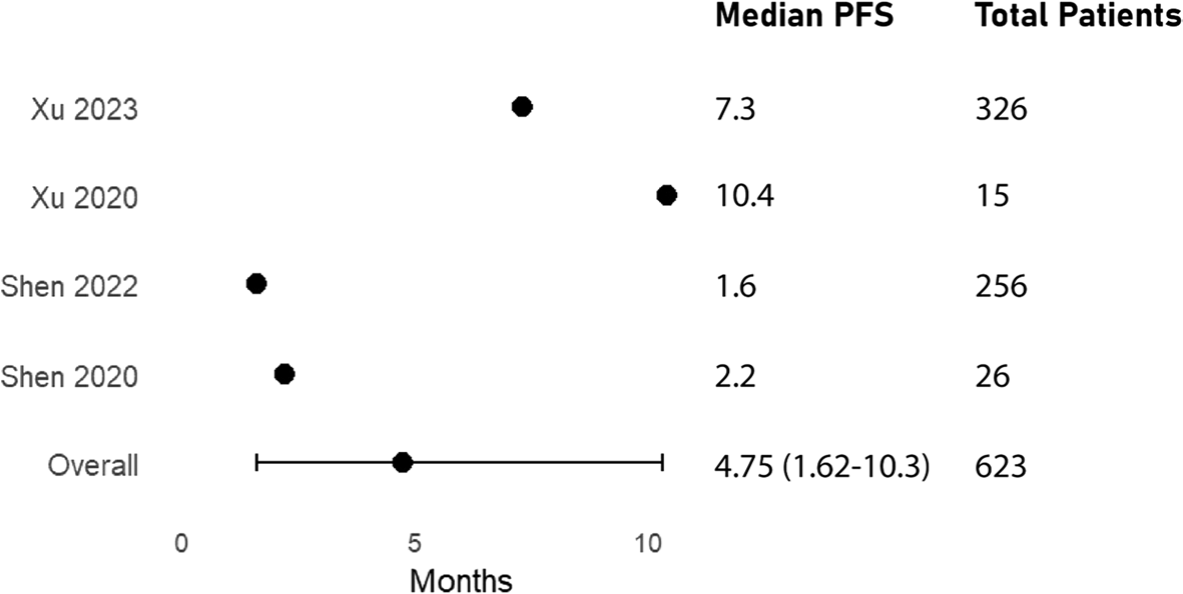
Pooled median progression-free survival (PFS) for patients receiving tislelizumab

The median OS data from three studies were pooled using the ‘meta median’ package in R. The combined median OS for patients treated with tislelizumab was 8.6 months (95% confidence interval [CI]: 4.8–17.2) (Fig. 3). Similarly, PFS data from all four studies were synthesized, yielding a pooled median PFS of 4.75 months (95% CI: 1.62–10.3) (Fig. 4).

**Fig. 5:**
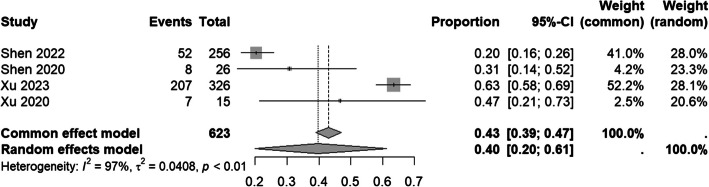
Pooled objective response rate (ORR) for patients receiving tislelizumab

**Fig. 6:**
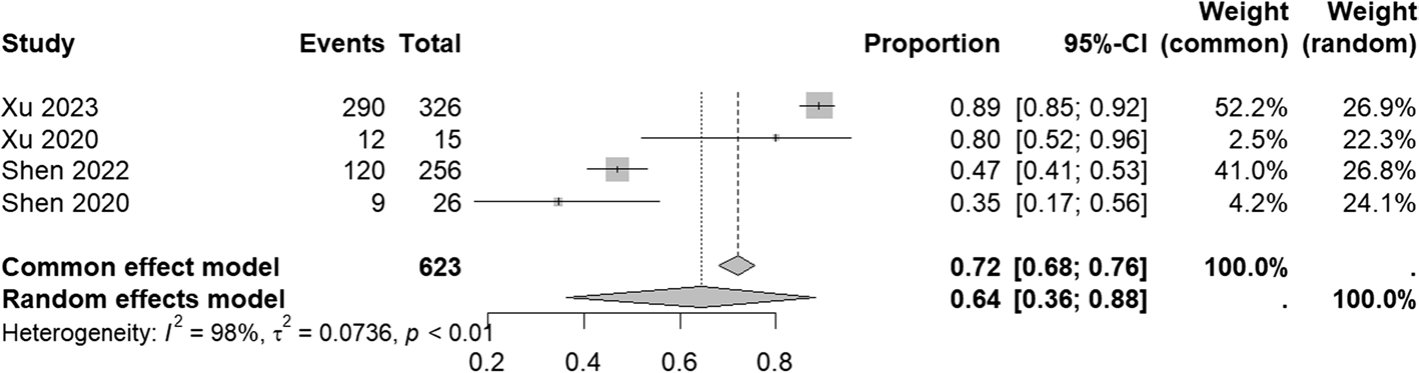
Pooled disease control rate (DCR) for patients receiving tislelizumab

The ORR was reported in all included studies. A random-effects model was applied due to significant heterogeneity (I^2^ = 97%). The pooled ORR for patients treated with tislelizumab was 0.40 (95% CI: 0.20–0.61) (Fig. 5). The DCR was also pooled using a random-effects model (I^2^ = 98%), resulting in an overall DCR of 0.64 (95% CI: 0.36–0.88) (Fig. 6). The high heterogeneity may be attributed to patient characteristics and treatment regimen variations.

**Fig. 7:**
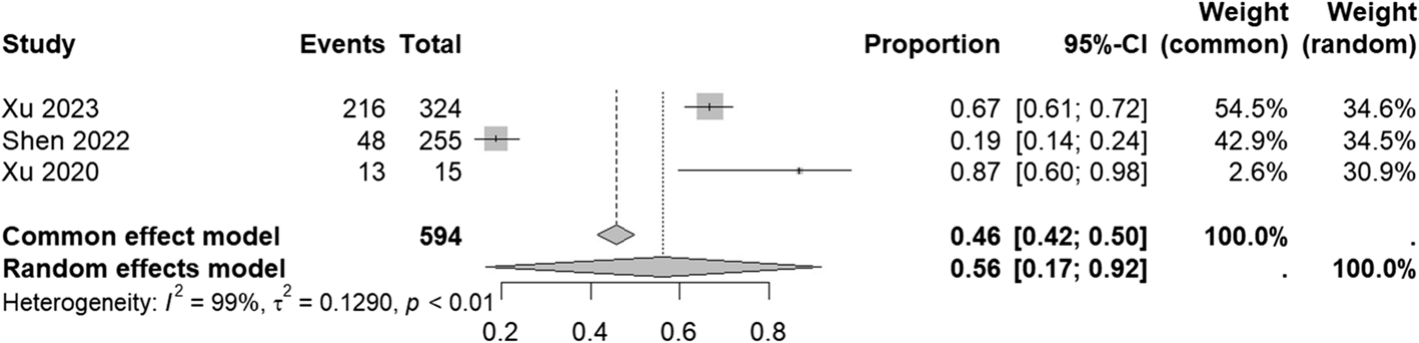
The pooled incidence of grade 3 or higher adverse events in patients receiving tislelizumab

**Fig. 8:**
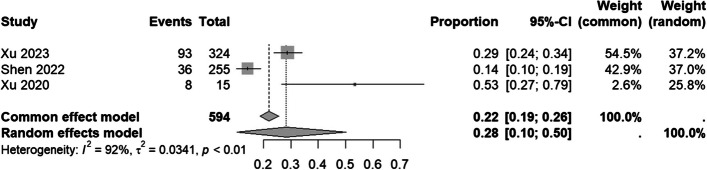
The pooled incidence of serious adverse events in patients receiving tislelizumab

In the safety analysis of single-arm studies, the incidence of grade 3 or higher adverse events was reported in three studies. The pooled incidence was 0.56 (95% CI: 0.17–0.92), with significant heterogeneity observed (I^2^ = 99%) (Fig. 7). Serious adverse events were also analyzed, with a pooled incidence of 0.28 (95% CI: 0.10–0.50) (I^2^ = 92%) (Fig. 8). Common grade 3 or higher adverse events included hematologic toxicities (neutropenia, leukopenia, anemia, thrombocytopenia), gastrointestinal disorders (vomiting), metabolic abnormalities (hyponatremia), and biochemical alterations (elevated liver enzymes, hypothyroidism).

**Fig. 9:**
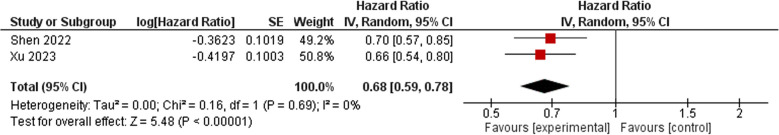
Forest plot of overall survival (OS) comparing tislelizumab versus control in RCTs

**Fig. 10:**
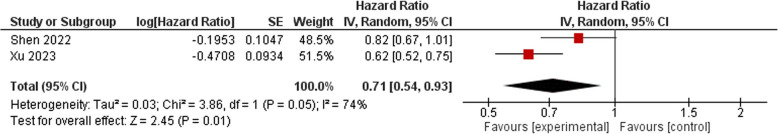
Forest plot of progression-free survival (PFS) comparing tislelizumab versus control in RCTs

For the two RCTs, comparative meta-analyses were performed between the tislelizumab group and the control group. The pooled HR for OS was 0.68 (95% CI: 0.59–0.78; *p* < 0.00001), indicating a 32% reduction in the risk of death for patients receiving tislelizumab (Fig. 9). There was no significant heterogeneity (I^2^ = 0%). For PFS, the pooled HR was 0.71 (95% CI: 0.54–0.93; *p* = 0.01), suggesting a 29% reduction in the risk of disease progression (Fig. 10). Moderate heterogeneity was observed (I^2^ = 74%).

**Fig. 11:**
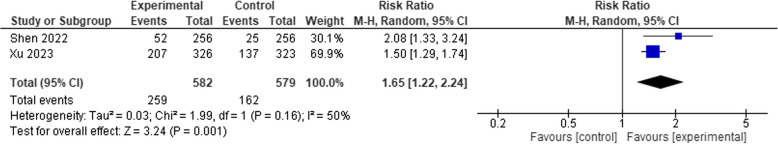
Forest plot of objective response rate (ORR) comparing tislelizumab versus control in RCTs

**Fig. 12:**
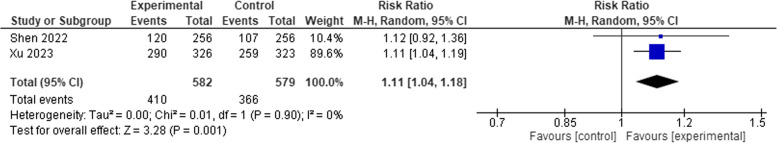
Forest plot of disease control rate (DCR) comparing tislelizumab versus control in RCTs

The ORR was significantly higher in the tislelizumab group, with a risk ratio (RR) of 1.65 (95% CI: 1.22–2.24; *p* = 0.001) (Fig. 11). Moderate heterogeneity was present (I^2^ = 50%). The DCR also favored the tislelizumab group, with an RR of 1.11 (95% CI: 1.04–1.18; *p* = 0.001) and no observed heterogeneity (I^2^ = 0%) (Fig. 12).

**Fig. 13:**
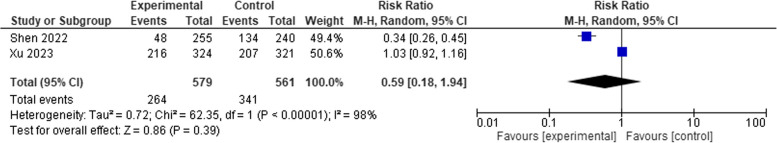
Forest plot of grade 3 or higher adverse events comparing tislelizumab versus control in RCTs

**Fig. 14:**
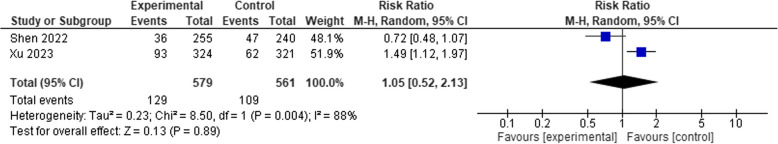
Forest plot of serious adverse events comparing tislelizumab versus control in RCTs

The incidence of grade 3 or higher adverse events did not differ significantly between the tislelizumab and control groups (RR = 0.59; 95% CI: 0.18–1.94; *p* = 0.39) (Fig. 13). Similarly, serious adverse events were comparable between groups (RR = 1.05; 95% CI: 0.52–2.13; *p* = 0.89) (Fig. 14). However, substantial heterogeneity was noted for both outcomes (grade 3 or higher adverse events: I^2^ = 98%; serious adverse events: I^2^ = 88%). The types of adverse events were consistent with those reported in the single-arm studies.

## Discussion

This systematic review and meta-analysis evaluated the efficacy and safety of tislelizumab in patients with advanced ESCC. The pooled results demonstrated that tislelizumab significantly improves OS and PFS compared to standard chemotherapy. Specifically, patients receiving tislelizumab experienced a 32% reduction in the risk of death (HR = 0.68) and a 29% reduction in the risk of disease progression (HR = 0.71). Additionally, the ORR and DCR were higher in the tislelizumab group, indicating enhanced tumor response. These findings align with previous studies on other PD-1 inhibitors in advanced ESCC, such as pembrolizumab and nivolumab [17, 18]. These have also shown significant improvements in survival outcomes and tumor responses. Notably, the combination of tislelizumab with chemotherapy enhances therapeutic efficacy. For example, patients receiving tislelizumab plus chemotherapy exhibited a median OS of 17.2 months, nearly double that of patients receiving tislelizumab monotherapy. This suggests a synergistic effect, potentially due to chemotherapy-induced immunogenic cell death enhancing the antitumor immune response mediated by tislelizumab [13–16]. Importantly, these benefits were observed regardless of PD-L1 expression, potentially expanding the applicability of tislelizumab to a broader patient population. Tislelizumab is designed to minimize macrophage interactions that may cause resistance to anti–PD–1 therapy. Early-phase trials showed its antitumor efficacy in solid tumors, including ESCC, with a safety profile similar to other anti–PD–1 antibodies [15].

The safety profile of tislelizumab was comparable to that of standard chemotherapy, with no significant increase in grade 3 or higher or serious adverse events. Common adverse events included hematologic toxicities (e.g., neutropenia, anemia), gastrointestinal disturbances (e.g., vomiting), metabolic abnormalities (e.g., hyponatremia), and immune-mediated events (e.g., hypothyroidism, pneumonitis). These adverse events were generally manageable with supportive care or dose adjustments and did not diminish the potential survival benefits of tislelizumab [19]. This safety profile is consistent with that observed in other cancers treated with tislelizumab, such as non-small cell lung cancer (NSCLC) and hepatocellular carcinoma (HCC) [9, 20].

The demographic characteristics observed in this study are consistent with previous findings. ESCC is more prevalent among men, with a male-to-female ratio of 4:1, and is commonly diagnosed in individuals around 65 years of age [21, 22]. The disease is widespread in Black and Asian populations, at rates of 85.5% and 75.4%, respectively [23]. This aligns with the study findings, which found that most participants were Asian.

This is the first meta-analysis dedicated explicitly to evaluating the efficacy and safety of tislelizumab in patients with advanced ESCC. While previous studies have investigated other immune checkpoint inhibitors in ESCC populations, no prior meta-analysis has comprehensively synthesized available clinical trial data focusing solely on tislelizumab [24]. This study fills an important gap by providing pooled estimates for key clinical outcomes across RCTs and non-RCTs. It offers clinicians and researchers a clearer understanding of tislelizumab's therapeutic potential and comparative benefit-risk profile. These findings also guide treatment decision-making and future research design in the evolving landscape of ESCC immunotherapy.

The FDA has approved tislelizumab plus platinum-containing chemotherapy for treating adult patients with advanced ESCC with a tumor PD-L1 expression of 1 or higher [25]. Recent National Comprehensive Cancer Network (NCCN) Guidelines for Esophageal and Esophagogastric Junction Cancers now also include tislelizumab in combination with chemotherapy as a preferred first-line treatment option for patients with ESCC and PD-L1 expression ≥ 1, based on the same clinical trials included in this meta-analysis [26]. This reflects the growing clinical acceptance of tislelizumab as a viable immunotherapy agent, and our findings provide timely and comprehensive evidence to support its integration into treatment practice.

Despite these promising results, several limitations of this review should be acknowledged. The small number of included studies and limited sample sizes may affect the robustness and generalizability of the findings. Including RCTs and non-randomized studies also introduces potential biases, such as selection bias and confounding variables inherent in non-randomized designs. Several analyses observed significant heterogeneity, likely due to variations in study designs, patient populations, treatment regimens (e.g., monotherapy vs. combination therapy), and baseline characteristics. The high heterogeneity may have influenced the pooled estimates and should be interpreted cautiously.

Potential effect modifiers, such as PD-L1 expression levels, prior treatments, and geographic differences, were not consistently reported across studies, limiting the ability to assess their impact on outcomes. Gaps in the information provided by the studies, such as long-term survival data and quality-of-life measures, highlight the need for further research. The search strategy was also limited to English-language publications, which may introduce language bias and exclude relevant studies published in other languages.

Future large-scale, high-quality RCTs are necessary to confirm these findings and to explore the optimal use of tislelizumab in advanced ESCC. Investigations into biomarkers predictive of response, standardization of PD-L1 testing, and assessments of long-term outcomes and quality of life would provide valuable insights. Understanding the reasons for heterogeneity and identifying potential effect modifiers could help tailor treatments to individual patients, enhancing efficacy and minimizing adverse events.

## Conclusion

Tislelizumab offers substantial clinical benefits in survival and tumor response for patients with advanced ESCC, as demonstrated by pooled OS, PFS, ORR, and DCR analyses from clinical trials, with significant improvements observed in randomized controlled trials. These findings underscore the clinical value of tislelizumab as an effective treatment option with an acceptable safety profile, supporting its incorporation into current treatment protocols for advanced ESCC. However, due to substantial heterogeneity and the limited number of available studies, further high-quality research is needed to confirm these results, inform evidence-based clinical guidelines, and address existing knowledge gaps. Policymakers and clinicians should consider these findings in decision-making while recognizing the need for additional data to optimize patient outcomes.

## Data Availability

No datasets were generated or analysed during the current study.

## Abbreviations

ESCC: Esophageal Squamous Cell Carcinoma
PD-L1: Programmed Death-Ligand 1
FDA: Food and Drug Administration
NSCLC: Non-Small Cell Lung Cancer
HCC: Hepatocellular Carcinoma
PRISMA: Preferred Reporting Items for Systematic Reviews and Meta-Analyses
PROSPERO: International Prospective Register of Systematic Reviews
ECOG PS: Eastern Cooperative Oncology Group Performance Status
RECIST: Response Evaluation Criteria in Solid Tumors
PD-1: Programmed Death-1
OS: Overall Survival
PFS: Progression-Free Survival
ORR: Overall Response Rate
DCR: Disease Control Rate
AE: Adverse Event
RoB 2: Revised Cochrane risk-of-bias tool for randomized trials
ROBINS-I: Risk Of Bias In Non-randomized Studies of Interventions
HR: Hazard Ratio
RR: Risk Ratio
CIs: Confidence Intervals
RCT: Randomized Controlled Trial
anti-PD-1: Anti-Programmed Death-1
TRAE: Treatment-Related Adverse Events
NCCN: National Comprehensive Cancer Network
